# Soldering

**Published:** 1897-07

**Authors:** 


ARTICLE XII.

### SOLDERING.

Cleanliness from beginning to end should always be looked after. It is very poor, if not dangerous, policy to attempt to solder a piece of work that has not first been boiled in pickle or in alum-water that the oxid which forms from the alloy in previous heatings shall be removed. Either that should be done or the plate and backing should be scraped to give a clean surface.

Dr. A. O. Hunt: There is one thought; the unequal expansion of platinum and porcelain is such that if a large piece of solder is laid over all, so that if at any time during the process of rotating the flame in, under and about the investment, it cannot come in direct contact with the platinum pins, there will not be a sudden expansion of the metal in the pins, causing the porcelain to check. A plate of heavier solder prevents the too rapid transition of heat to the metal underneath, and thus equalizes more nearly the expansion of the metal and porcelain. I prefer the bellows to the blow-pipe. There are features in the use of the former that I regard as important. With the blow-pipe in the mouth the head must be at a fixed distance from the flame nearly all the time. One hand is occupied in directing the blow-pipe, while in using the bellows both hands and the head are free, so that one is enabled to see any part of the work to better advantage. A continuous flame can be managed by the bellows with so little effort that it is a decided improvement over the mouth blow-pipe. One other point is with reference to the kinds of flame to be used. A fine reducing flame of the blow-pipe is rarely ever called for in joining the points of a crown or bridge. The brush or larger flame, which will cover the greater part of the piece to be soldered, is more desirable, as one is less likely to break the porcelain, the heat being more evenly distributed over a large area.

Dr. W. H. Taggart: Draw the solder to one point with the blow pipe flame, to melt and to evenly distribute the solder. The heat will go to the hottest part, and there is a tendency for all the solder, when it has become melted, to gravitate to one point, leaping parts that are already soldered properly. You have to rotate it to get it to stay at one point. It is much easier to do this with the blow-pipe than with anything else. The fewer pieces of solder you put on at one time the better you fuse it, and get it to flow into all places without forming pits or pockets. It would be an advantage to put on pieces as they melt than to put on all pieces at one time.

Dr. F. N. Brown: Crown and bridge-work are my food and dirnk; I scarcely study anything else. The first thing I do when I procure any teeth from the dental depot, is to remove every particle of wax and allow none to come in contact with my work while in process of construction. I use "Parr's wax flux" in waxing up and holding parts together till the piece is invested. It is then placed over a Bunsen heater and "Parr's wax flux" burns out, leaving the piece fluxed. I then sprinkle it with a little dry powdered borax and place enough solder on to cover the pins of the teeth. I then proceed to heat the investment and solder up, adding one piece of solder at a time. I never remove my piece of work to a soldering block, or anything else, but let it remain on the Bunsen heater while soldering up.

Some breakages come from backings being so positioned as to put strain on the porcelain in cooling. I think most breakages come from allowing the porcelain facings to touch, and that from expansion the facings are cracked. In soldering, I never use the mouth blow-pipe; I prefer a blow pipe on a stand, so that I can keep a foot-bellows or compressed air apparatus going, then having the flame constantly directed on the work. In this way there is no cessation of heat at any time. The use of "Parr's wax flux" in bridge-work cannot be overvalued.

Dr. B. J. Cigrand: I desire to say a few words in reference to Dr. Peck's paper It is a paper that I think will do both the older and younger members a great deal of good. Investment is only holding the parts in apposition and position while soldering; in single small pieces, where the parts can be held in apposition by wire or otherwise, investment is not necessary. Some years ago I learned a lesson in soldering under somewhat exciting circumstances. When I first started to practice dentistry, one of my first cases called for the application of a bridge to restore the teeth. While I had the bridge and large investment on the fire, my attention was directed to the fact that I had some errand to do down town. I went down town to one of the dental depots, unintentionally left my investment and bridge on the fire at the office, and did not return for about two hours. While I was down town it occurred to me that I had left my bridge and investment on the fire, and you may be sure it did not take me very long to catch the first car. When I reached my office I believed that the bridge was spoiled, and as I am somewhat of an optomist, I tried to make the best of it. I made up my mind that the bridge was spoiled. I saw that it was red hot and thoroughly radiated, and after using the blow-pipe a little, the solder blowed away as so much cream.

I was determined, in the event of a failure, to have the personal pleasure of having brought it about, executed my intent, little thinking that an underlying principle in soldering would be revealed. I threw the case in powdered plaster of Paris to cool, and, on disinvesting the case, I found I had made one of the best solders in my life; the secret of which is: use the blow-pipe very little and get the case good and hard. I hardly ever use a blow-pipe except in large bridges. I use the Haskell-Bunsen burner for the entire operation, never employing a blow-pipe in the production of what we call barrel or telescope crown.

The globulating of the solder arises from the fact that the parts are not thoroughly heated, or there is present some foreign matter on the surface. Most solders are composed of several base metals. If the solder is globulated, and we still pour on the heat, the result is the zinc or brass is burnt out. The low-fusing metal is burnt out because you have poured a constant stream of fire on the globulated solder. This results in your having pure copper or silver remaining. You have in consequence a high-fusing solder on the gold. You constantly keep on the annealing point, and there is a hole burnt in the gold. This shows the utter disadvantage of using the blow-pipe to much extent. To prevent the solder from flowing in the direction where not wanted, I employ a paste of whiting and rouge.

When I make a barrel or telescope crown, after I have made the band and readjusted the cusps, and invert it and proceed to solder it to the band, the band may, while in the flame, become unsoldered. If the stove blacking or whiting, whichever you prefer, is used on the joint of the band you can solder the cusps full of gold, and there will be no unsoldering or opening up of the band. I have recently resorted to the method of filing all my solders as they come from the dental depots into almost a dust; to eliminate all of the steel, etc., from the filings, I pass through it a large magnet. This way your solders will melt much quicker since you create a greater surface for the flame to play on. With reference to holding the crown in the flame and the liklihood of the solder jumping out of position on account of the puckering up of the borax, if we take the crown and use gum-arabic, making it into a mucilage over the borax, we can force or hold the solder wherever we like and it will stick there.

Dr. J. H. Woolley: Borax in the process of melting has a tendency to move the solder. If the borax is melted previous to its use and ground finely, it will not interfere with the solder, so that that process makes the placing of the solder just where it belongs, or where it is needed.

Dr, Peck: A crown can be as easily contoured in the Bunsen flame as if invested. The solder can be melted on one side to any extent desired without disturbing that on the other side.

The idea that in soldering large cases very small pieces of solder should be melted into the crevices, etc., after which more is added from time to time till the case is completed, I consider one of the greatest mistakes one can make in doing this work. What is the use of occupying the extra time necessary to permit the case to partially cool, to place more solder, and to reheat, and to repeat several times till the soldering is completed, when all can be done in most cases with one heating. All that is neccssary is care in the preparation of the case for soldering; the crevices must be thoroughly cleansed and fluxed, and the case thoroughly heated from the under side. If this preparatory work is not thoroughly done we must not expect desirable results.

I do not think the teeth or facings in contact, one with the other, are any more liable to break than when they are spaced—providing the case is evenly and thoroughly heated. Many times I have soldered large cases in which the teeth were in close contact one with the other, in fact, in some instances, even jointed together, and I haven't gotten into trouble. I can't help believing that the predominating cause of the breakage of teeth and facings is the untimely heating of the pins.—*Dental Review.*

